# Coumarin: A Natural, Privileged and Versatile Scaffold for Bioactive Compounds

**DOI:** 10.3390/molecules23020250

**Published:** 2018-01-27

**Authors:** Angela Stefanachi, Francesco Leonetti, Leonardo Pisani, Marco Catto, Angelo Carotti

**Affiliations:** Dipartimento di Farmacia-Scienze del Farmaco, Università degli Studi di Bari “Aldo Moro”, via E. Orabona 4, I-70125 Bari, Italy; angela.stefanachi@uniba.it (A.S.); francesco.leonetti@uniba.it (F.L.); leonardo.pisani@uniba.it (L.P.); angelo.carotti@uniba.it (A.C.)

**Keywords:** coumarin, aromatase inhibitors, cholinesterase inhibitors, monoamine oxidase inhibitors, multitarget ligands

## Abstract

Many naturally occurring substances, traditionally used in popular medicines around the world, contain the coumarin moiety. Coumarin represents a privileged scaffold for medicinal chemists, because of its peculiar physicochemical features, and the versatile and easy synthetic transformation into a large variety of functionalized coumarins. As a consequence, a huge number of coumarin derivatives have been designed, synthesized, and tested to address many pharmacological targets in a selective way, e.g., selective enzyme inhibitors, and more recently, a number of selected targets (multitarget ligands) involved in multifactorial diseases, such as Alzheimer’s and Parkinson’s diseases. In this review an overview of the most recent synthetic pathways leading to mono- and polyfunctionalized coumarins will be presented, along with the main biological pathways of their biosynthesis and metabolic transformations. The many existing and recent reviews in the field prompted us to make some drastic selections, and therefore, the review is focused on monoamine oxidase, cholinesterase, and aromatase inhibitors, and on multitarget coumarins acting on selected targets of neurodegenerative diseases.

## 1. Introduction

This review is focused on the design, synthesis, bio-pharmacological evaluation, and data analysis of a large number of coumarin derivatives, mainly developed in our group, as monoamine oxidase (MAO), cholinesterase (ChE), and aromatase (AR) inhibitors, and as multitarget agents addressing neurodegenerative diseases (NDs). Coumarin derivatives studied by other groups will be also taken into account, to expand the analysis of the structure–activity relationships (SARs), but not in an extensive/comprehensive way, most of them being already quoted in a recent book [1] and exhaustive reviews [2,3,4,5,6,7,8,9,10,11,12,13,14,15,16,17].

First, a general overview of coumarins will be presented, followed by a discussion on the design, synthesis, and biological evaluation as enzyme inhibitors and multitarget ligands of suitably functionalized coumarins.

### 1.1. General Overview on Coumarins

Coumarin **1** was first isolated in 1820 by Vogel [18] from tonka beans (*Dipteryx odoranta* Wild; *Fabaceae* family) called also *Coumarou*, a vernacular French name. Since then, isolation, structural characterization, synthesis, and biological activity of thousands of natural coumarins from plants, bacteria, fungi [19,20,21], and chemical synthesis [22], have been reported.

**Figure 1:**
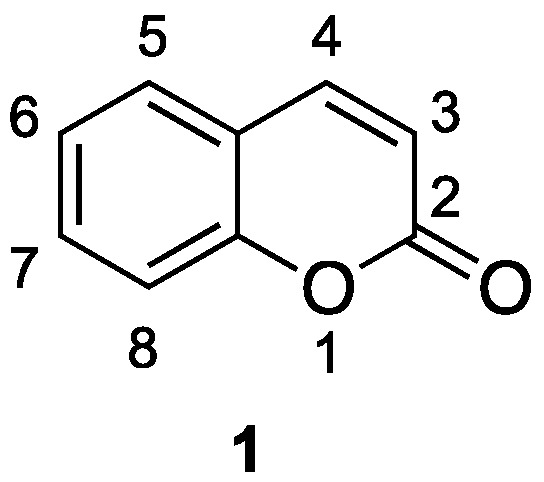
Structure and numbering scheme of coumarin **1**.

**Figure 2:**
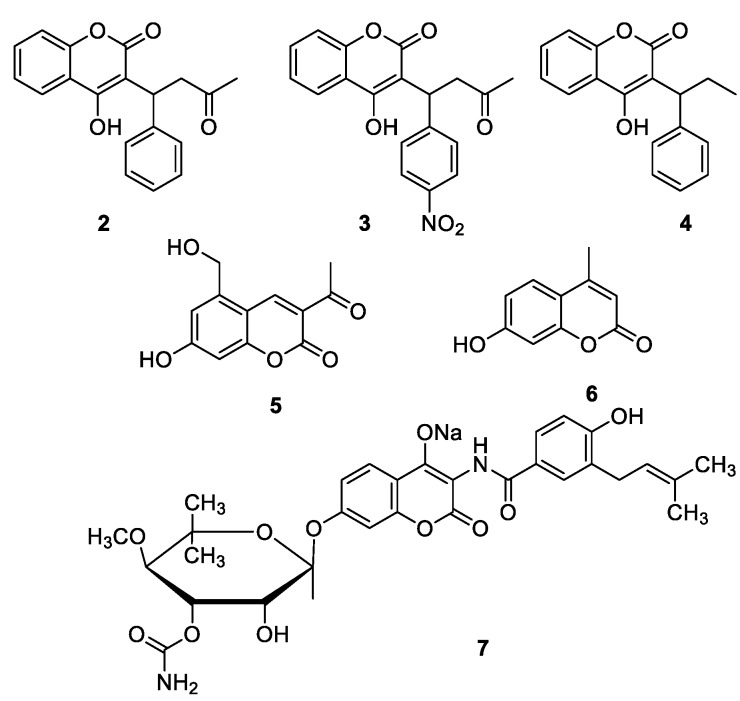
Structures of some coumarin drugs: the anticoagulants warfarin **2**, acenocoumarin **3**, and phenprocoumon **4**, the choleretics armillarisin A **5** and hymechromone **6**, and the antibiotic novobiocin **7**.

Coumarin **1**, whose structure and numbering scheme are illustrated in Figure 1, is characterized by a 2*H*-chromen-2-one (1,2-benzopyrone, or 2*H*-1-benzopyran-2-one) oxa-heterocycle, and has been largely studied since its skeleton is present in many biologically active agents. Coumarin and some of its derivatives have become drugs, as the anticoagulants warfarin **2**, acenocoumarin **3**, and phenprocoumon **4**, all acting as vitamin K antagonists, the choleretics armillarisin A **5** and hymecromone (umbelliferone) **6**, and the antibiotic novobiocin **7** (Figure 2), which is a potent inhibitor of bacterial DNA gyrase (GyrB).

### 1.2. Biosynthesis and Metabolism of Coumarins

**Figure 3:**
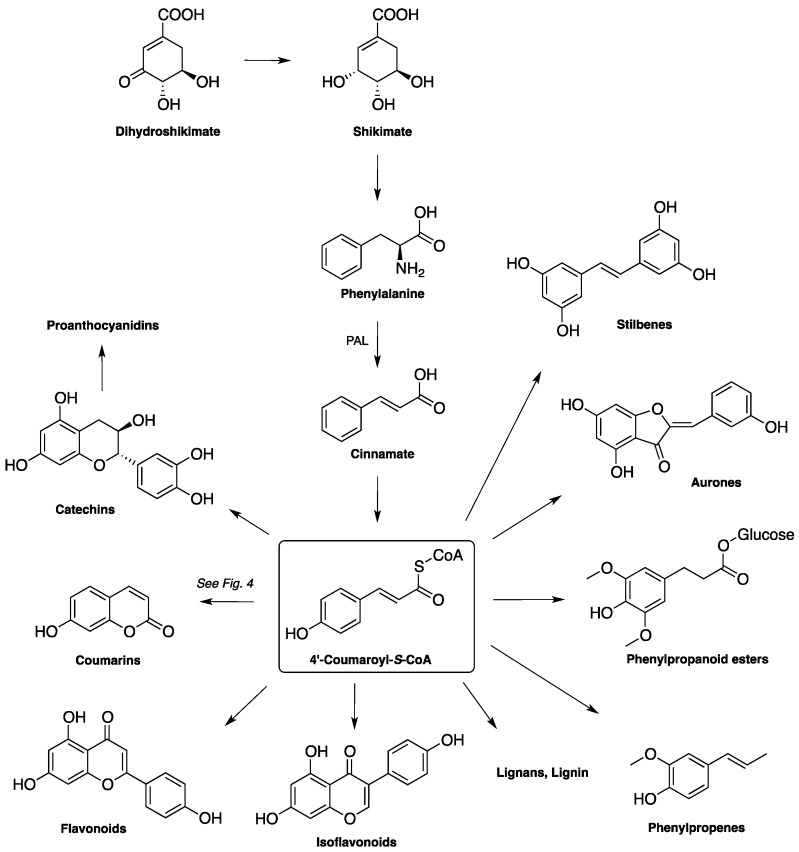
General biosynthetic pathway leading to phenylpropanoids (adapted from Vogt, 2010) [23].

Naturally occurring coumarins are synthesized through the general biosynthetic pathway, leading to phenylpropanoids, as illustrated in Figure 3 [23].

**Figure 4:**
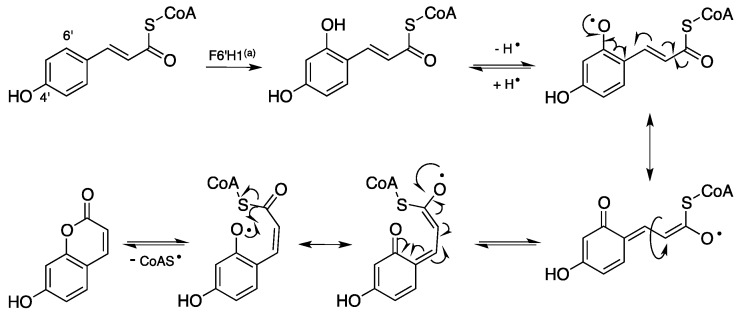
Proposed radical mechanism of 7-hydroxycoumarin biosynthesis from 4′-coumaroyl-*S*-CoA (adapted from Kai et al., 2008) [24]. ^(a)^: 2-oxoglutarate-dependent dioxygenase F6′H1.

Phenylalanine, which is formed through the shikimate biosynthetic pathway, is converted by phenylalanine ammonia lyase (PAL) into the *trans*-cinnamic acid, which in turn originates the central metabolite 4′-coumaroyl-*S*-CoA. This crucial intermediate can then be transformed in many classes of phenylpropanoids, reported in part in Figure 3. The biosynthesis of coumarin from 4′-coumaroyl-*S*-CoA central metabolite takes place through the 6′-hydroxylation, the *trans* > *cis* isomerization of the exocyclic double bond, and the final lactonization/cyclization reaction. A first and crucial step of the biosynthesis is the 6′ (*ortho*) hydroxylation catalyzed by the 2-oxoglutarate-dependent dioxygenase F6′H1. The radical mechanism of the isomerization and lactonization reactions, illustrated in Figure 4, has been proposed by Kai et al. in 2008 [24].

**Figure 5:**
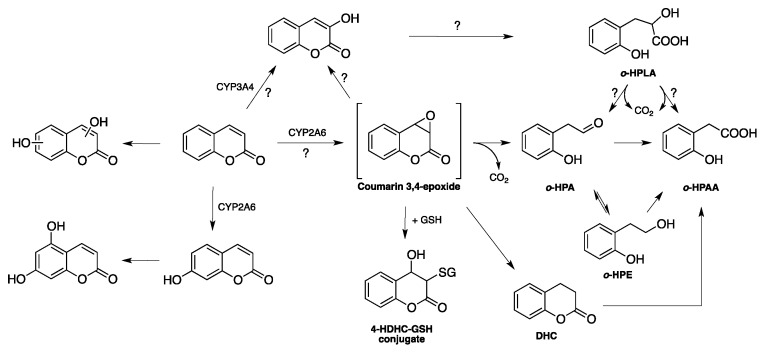
Representative pathways of coumarin metabolism (adapted from Lake, 1999) [25].

As far as the metabolism of coumarin is concerned, two main pathways have been disclosed: the 7-hydroxylation, and the opening of the lactone ring with the loss of carbon dioxide (Figure 5). This last reaction takes place on the coumarin 3,4-epoxide intermediate formed in the first step of the metabolic pathway. Actually, under aqueous conditions, this epoxide liberates carbon dioxide to form *ortho*-hydroxyphenyl acetaldehyde (*o*-HPA) that can be further metabolized to the corresponding acid (*ortho*-hydroxyphenyl acetic acid, *o*-HPAA) and alcohol (*ortho*-hydroxyphenyl ethanol, *o*-HPE). The 3,4-epoxide may undergo a nucleophilic attack by glutathione to form the 4-HDHC-GSH (4-hydroxy-3,4-dihydrocoumarin-3-mercapturic acid), and may be transformed into 3-hydroxycoumarin and then into *ortho*-hydroxyphenyl lactic acid (*o*-HPLA), which finally affords *o*-HPA and *o*-HPAA. As indicated in Figure 5, other possible metabolites of coumarin are the 3,4-dihydrocoumarin (DHC) and hydroxylated coumarins at positions 4, 5, 6, and 8, which are formed at a much lower extent than at position 7. Question marks on some pathways reported in Figure 5, indicate reactions, and/or reaction mechanisms, which are still under investigation.

A pivotal role in the metabolic transformation of coumarin is played by some cytochromes P450 (CYPs). CYPs are iron-containing enzymes (hemoproteins) that catalyze redox reactions of a variety of endo- and xenobiotics, including drugs, food nutrients, and environmental pollutants [26,27]. CYPs play a key role in bioactivation, toxification, and detoxification processes of xenobiotics, thus regulating metabolism and toxicity of many drugs and chemicals [28]. CYP1, CYP2, and CYP3 are the main CYP families responsible for the oxidative biotransformation of the majority of drugs. Among human CYPs, one relevant drug-metabolizing enzyme is CYP2A6 [29]. CYP2A6 is the major enzyme involved in the key, and prevalent, metabolic transformation of coumarin to 7-hydroxycoumarin (Figure 5) in human liver microsomes [30]. A reduced CYP2A6 activity, deriving also from the large gene multiplicity and polymorphism observed for CYP2A6, may favor alternative metabolic pathways of coumarin, such as the one leading to 3-hydroxycoumarin under the catalysis of CYP3A4. It has been suggested that a high level of 3-hydroxycoumarin promotes the formation of the cytotoxic product *o*-HPA (Figure 5), that therefore may be responsible/co-responsible for the toxicity arising from coumarin. Coumarin is effectively used in the pharmacological treatment of lymphedema, a chronic progressive and disabling disease affecting millions of people worldwide. Unfortunately, this cheap and efficient drug has been banned in some countries for possible hepatotoxic effects, unquestionably proven, indeed, in rats and mice, and more rarely, in humans, as underlined earlier. However, in the light of the previous findings, lymphedema patients with low active CYP2A6 should be identified and not be treated with coumarin, since they would metabolize it via the cytotoxic pathway leading to *o*-HPA. The preliminary identification (phenotyping) of these patients should permit a safer use of the efficacious and cheap coumarin on all the other lymphedema patients with normally functioning CYP2A6 enzyme [31]. This challenging hypothesis may be further substantiated, since it is in contrast with the results obtained from a kinetic study of the 3,4-epoxide coumarin formation [32] (see Section 1.3).

### 1.3. Toxicity of Coumarins

Dietary exposure to coumarins is quite high, as they are found in vegetables, fruits, seeds, nuts, coffee, tea, and wine. It is estimated that the average Western diet may contain approximately 1g/day of benzopyrones, chiefly coumarins and flavonoids [25]. Therefore, extensive research on the biological, pharmacological, and also toxicological properties of coumarins has been carried out. The most significant results of the diverse toxicity studies have been collected in some reviews. Metabolism, toxicity, and carcinogenicity studies have been reviewed [33] from studies on the safety for humans of coumarins present in foodstuffs, and in fragrances for cosmetic use. In that review, the authors concluded that exposure to coumarins from food and/or cosmetic products poses no health risk to humans. Conversely, other papers point out some significant toxicity of coumarin and some coumarin derivatives. In fact, hepatotoxic effects have been found in hepatocytes of different species, including humans [34,35,36]. In another interesting paper [37], it has been pointed out that cytotoxic effects of coumarins are metabolism- and species-dependent, and as a consequence, rat models cannot be used to evaluate a possible toxicity of coumarin in humans. Indeed, an in vitro kinetic study of *o*-HPA formation, and in particular, the large quantities of coumarin required for *o*-HPA production in human liver microsomes, suggested that humans are unlikely to produce toxicologically relevant concentrations of this metabolite, coming from the highly reactive 3,4-coumarin epoxide and 3-hydroxycoumarin, because of the relatively low level of coumarin exposures [32]. Studies in zebrafish embryos suggested for coumarin and warfarin teratogen and lethal effects, but at high doses [38]. Developmental toxicity was claimed for coumarin and hydroxycoumarins [39].

Interestingly, recent studies based on human data indicated a tolerable dose intake (TDI) of coumarin equal to 0.1 mg/kg body. This dose must not be exceeded to avoid toxic effects. Indeed, during Christmas season in Germany, the consumption of cassia cinnamon has resulted in such a high dose that the TDI of coumarin was often reached, thus elevating the risk for hepatotoxic and carcinogenic effects [40].

### 1.4. Pharmacological Activities of Coumarins

The numerous pharmacological activities of coumarins depend on their core structure (e.g., simple coumarins, fused polycyclic coumarins, bis-coumarins) and on the substitution pattern. Among the most studied pharmacological activities, it is worth mentioning antibacterial [1,22], antitubercular [13], antifungal [41], antiviral [42], antimutagenic [43], antioxidant [44], scavenging of reactive oxygen species (ROS) [45], anti-inflammatory [46], antithrombotic [47] anticancer [48], anticoagulant [49] activities, and cyclooxygenase [50], lipooxygenase [51], cholinesterase (ChE) and monoamine oxidase (MAO) inhibitory activities, CNS stimulant [52], vasodilator [53], and cytotoxic [54] effects.

**Figure 6:**
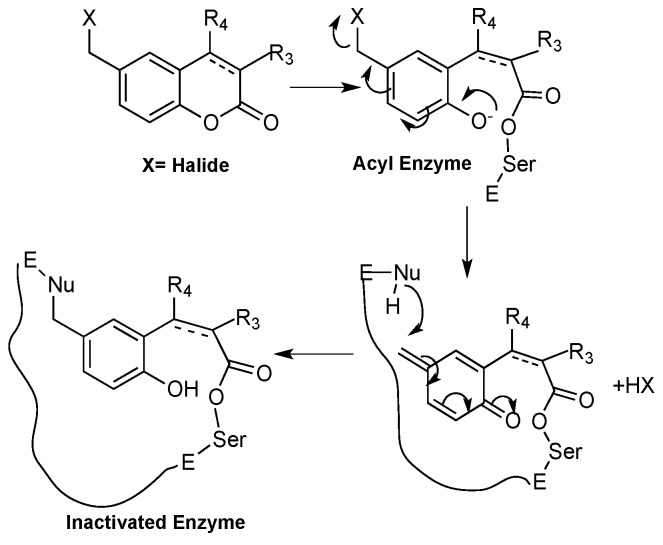
Mechanism of inhibition of serine proteases by 6-halomethyl-3,4-dihydrocoumarins (suicide substrate/inhibitor). Adapted from Pochet et al., 2004 [56].

The numerous bio-pharmacological activities of coumarin surely depend on the peculiar chemical structure and physicochemical properties of its oxaheterocyclic ring, that allows easy binding to many protein targets. The 2*H*-chromen-2-one ring is planar, aromatic, and lipophilic, and therefore, is able to interact with biological counterparts, mainly lipophilic binding sites, by establishing strong hydrophobic, and more often, π–π stacking interactions with aromatic amino acids, i.e., Phe, Tyr, and Trp. Positively charged amino acids might bind coumarins through strong cation–π interactions. Moreover, the lactone group of the coumarin confers to the molecule the ability to make strong polar binding, like hydrogen bonds and dipole–dipole interactions, and, sometimes, to acylate target proteins, as claimed for the covalent mechanism of inhibition of some enzymes. Actually, enzymes with esterase activity can also open the lactone ring, and the compounds resulting from this hydrolysis may be the responsible for the observed biological activity. In this case, coumarins act like pro-drugs, being bioactivated to liberate the true active metabolites. This mechanism of action has been proposed for the inhibition of carbonic anhydrase, which also has esterase activity, by a series of natural coumarins [55]. The lactone ring of coumarins and 3,4-dihydrocoumarins may acylate also serine proteases, like human leucocyte elastase (HLE) [56] and kallikrein [57]. Actually, in both studies, suicide inhibitors have been designed by placing a halomethyl group at position 6 as the second electrophilic center for the attack of a nucleophilic group of the enzyme. According to the mechanism delineated in Figure 6, after the attack of the catalytic serine at the lactone carbonyl, an electronic rearrangement takes place on the intermediate acyl-enzyme, with the elimination of the halide and the formation of highly conjugated exocyclic double bond that undergoes an easy nucleophilic addition of a nucleophilic group of the enzyme, which is ultimately trapped by two covalent bonds in a final inactivated form.

Whatever the mechanism of action of coumarins, the strength of their binding to the target is augmented by additional interactions involving the substituents present on the coumarin scaffold. Type of substituents and substitution pattern determine, besides the overall binding energy and potency, the selective interactions of coumarin derivatives with specific targets, ultimately establishing their bio-pharmacological profile.

## 2. Synthetic Strategies for the Preparation of Coumarins

As anticipated in Section 1.1., the coumarin scaffold characterizes a large variety of biologically active natural products, pharmaceuticals, agrochemicals, and polymeric [2] and optoelectronic [3] materials. For this reason, huge and continuous efforts have been devoted to the development of new synthetic pathways and protocols to more easily perform the key cyclization reaction to the heterocyclic ring and its regioselective derivatization. Indeed, large emphasis has been placed on the development of more efficient and greener synthetic approaches to produce properly designed coumarin derivatives. As a result, the increasing use of new/enabling technologies, such as microwaves and ultrasound, new catalysts, and greener solvents (or even solvent-free reactions) in recent years has made much simpler the access to coumarin derivatives. Among the numerous reactions proposed, those based on transition metal catalysts are the most exploited for high-yield syntheses of coumarins under generally mild experimental conditions. These, and similar reactions, have been recently summarized in comprehensive reviews [58,59]. The various retrosynthetic approaches developed for the preparation of coumarin derivatives, and, in particular, new methods leading to coumarins through the classical Pechmann condensation reaction, have been collected and efficiently illustrated in a general review on anticancer coumarins [5]. Therefore, here below, only selected synthetic methods published since the late 2015 will be briefly reported.

The classical and most used methods to access coumarin derivatives e.g., the Pechmann [60,61] and Knoevenagel [62] reactions, are still the object of numerous investigations, mainly aimed at (a) improving yields; (b) using only recyclable and/or green catalysts and solvents; and (c) developing easy and straightforward work-up procedures. Solvent-free reactions and sonochemistry have been also evaluated as alternative, enabling technologies to reach the objectives (a–c) delineated above.

The newly published synthetic approaches for the synthesis of coumarins reported below are grouped according to their homogeneity and similarity.

### 2.1. Regioselective Synthesis of 3-Substituted Coumarins

**Scheme 1:**
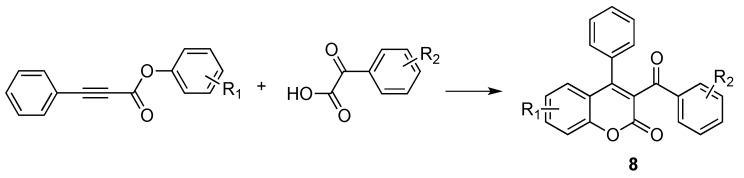
Synthesis of 3-aroylcoumarins from alkynoates.

(A) Synthesis of 3-aroylcoumarins **8** from the reaction of alkynoates with α-keto acids (Scheme 1).

**Scheme 2:**
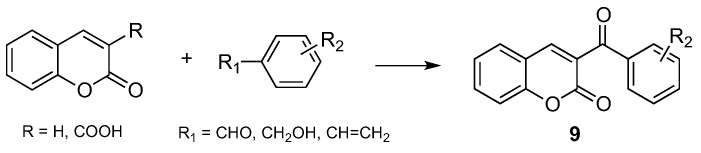
Synthesis of 3-aroylcoumarins from coumarin or coumarin-3-carboxylic acid.

Yan and coll. [63] reported a convenient silver-mediated radical cyclization method for the synthesis in high yields of coumarin derivatives. The reaction is carried out with dipotassium peroxodisulfate, silver nitrate in water/acetonitrile in about 24 h, at 60 °C, in a sealed tube under an inert atmosphere.

(B) Synthesis of 3-aroyl coumarins **9** from the reaction of coumarins, or coumarin-3-carboxylic acids with benzaldehydes, benzyl alcohols, and styrenes (Scheme 2).

**Scheme 3:**
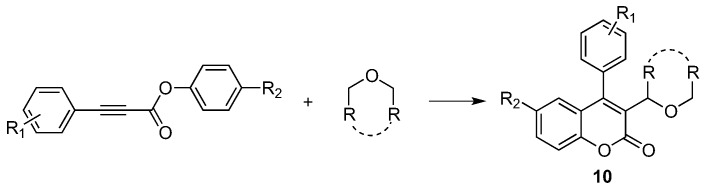
Synthesis of 3-substituted coumarins from alkynoates.

A metal-free, radical reaction has been performed with tert-butylhydroperoxide in water, chlorobenzene, for 20 h and at 100 °C, and in sealed tube [64]. Interestingly, the reaction takes place in good to high yields, also by using benzyl alcohols and styrenes as carbonyl surrogates.

(C) Synthesis of coumarins substituted at position 3 with alkoxy groups or saturated oxaheterocycles **10** (Scheme 3).

**Scheme 4:**
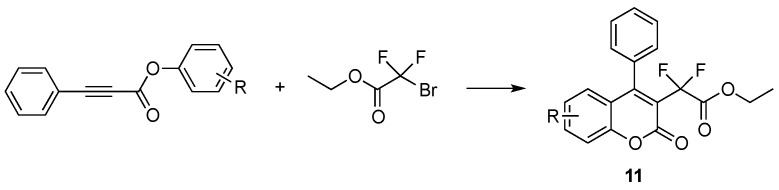
Synthesis of 3-difluoroacetylated coumarins from alkynoates.

The reaction was performed in experimental conditions close to the ones reported in (A), i.e., with *tert*-butylhydroperoxide, tris(bipyridine)ruthenium(II) dichloride hexahydrate in decane, acetonitrile, at room temperature, under ultrasound irradiation and inert atmosphere [65].

(D) Synthesis of 3-difluoroacetylated coumarins **11** from the reaction of alkynoates with ethyl bromodifluoroacetate (Scheme 4).

The visible light-mediated radical cyclization was carried out with fac-Ir(2-phenylpyridyl)_3_ and potassium carbonate in DMF, for 8 h, at ambient temperature under an inert atmosphere [66]. The reaction allows the direct formation of two C–C bonds via a proposed tandem radical cyclization process, as for reaction (A). Coumarins **11** were obtained in good yields.

Knoevenagel reaction

**Scheme 5:**
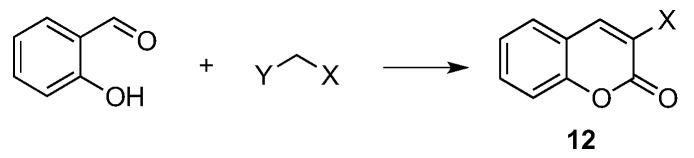
Synthesis of 3-substituted coumarins from salicylaldehyde.

Knoevenagel condensation leading to coumarins has been recently reviewed [67].

(A) Synthesis of 3-substituted coumarins **12** catalyzed by potassium phthalimide (KPhT) (Scheme 5).

An expeditious, efficient and green procedure for the KPhT catalyzed synthesis of 3-carboxy and 3-cyanocoumarins in high yields has been reported [68]. The reaction of salicylaldehydes with active methylene compounds (X–CH_2_–Y) was carried out under mild conditions in water at room temperature for 0.5–4 h.

(B) Synthesis of 3-substituted coumarins catalyzed by MgFe_2_O_4_ nanocatalyst under ultrasound irradiation.

Knoevenagel condensation between various salicylaldehydes and 1,3-dicarbonyl compounds, by using MgFe_2_O_4_ nanoparticles as an efficient catalyst under solvent-free conditions and ultrasound irradiation, has been reported [69]. High yields, simple work-up procedure and short reaction times are further advantages of the proposed protocol.

### 2.2. Synthesis of Coumarins by the Pechmann Reaction

Pechmann condensation reaction, one of the most studied synthetic approaches for the synthesis of coumarins, is still the object of numerous investigations. Some of the most recently proposed procedures are briefly reported herein.

(A) γ-Fe_2_O_3_@HAp-Ag NPs as catalyst in the synthesis of coumarins.

An easily prepared catalyst, that is Ag supported on the hydroxyapatite-core–shell magnetic γ-Fe_2_O_3_ nanoparticles (γ-Fe_2_O_3_@HAp-AgNPs) efficiently catalyzed the Pechmann reaction. The magnetically recyclable catalyst gave the desired coumarins in high yields, in eco-friendly experimental conditions, and with an easy work-up procedure [70].

(B) FeCl_3_-catalyzed synthesis of coumarins.

Moderate to excellent yields have been obtained by the classical Pechmann reaction of activated phenols with β-ketoesters by using 10% mol of FeCl_3_·6H_2_O as catalyst [71].

(C) Molybdate sulfuric acid-catalyzed synthesis of coumarins.

Title sulfuric acid has been used as a new and efficient catalysis for the Pechmann reaction carried out in water–dioxane at 80 °C, and affording the expected coumarins in good yields [72].

(D) SnCl_4_ grafted on silica as catalyst for the synthesis of coumarins.

The heterogeneous catalyst promotes the coumarin formation under free solvent conditions at 120 °C in moderate to high yields [73].

(E) Lewis acid grafted sulfonated carbon@titania composite as an efficient catalyst for the synthesis of coumarins.

Lewis acid C@TiO_2_–SO_3_–SbCl_2_ catalyst, prepared from carbon@titania composite showed excellent efficiency in the catalysis of the Pechmann reaction, which was carried out in high yield without solvents, at 60 °C [74].

(F) Synthesis of coumarins catalyzed by sawdust–SO_3_H.

The biodegradable and recyclable solid sawdust–SO_3_H catalyst afforded coumarins via the Pechmann reaction at 110 °C, in high yields, within a short time (<1 h), no solvent, and easy work-up procedure [75].

(G) l-Ascorbic acid as promoter of the synthesis of coumarins.

l-Ascorbic acid (vitamin C) was proven a green and efficient promoter of the high-yielding synthesis of coumarins and flavones under solvent free conditions at high temperature (180 °C) but in a short time (<30 min) [76].

(H) Ionic liquid-catalyzed synthesis of coumarins.

1,3-Disulfonic acid imidazolium hydrogen sulfate has been used as an effective and reusable ionic liquid catalyst under solvent-free conditions. High yields were afforded at 70 °C, in short time (< 30 min), and under solvent-free conditions [77].

### 2.3. Miscellaneous Reactions for the Synthesis of Coumarins

**Scheme 6:**
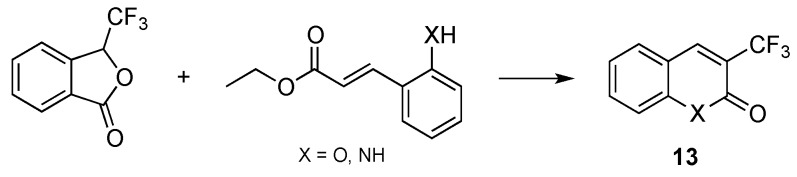
Synthesis of 3-trifluoromethyl coumarins and carbostyryls from cinnamic esters.

(A) Regioselective synthesis of 3-trifluoromethyl coumarins and carbostyryls **13** from *ortho*-hydroxy and *ortho*-amino cinnamic esters (Scheme 6).

The reaction was accomplished in high yields with the Togni reagent as the CF_3_ source, by using copper(l) iodide as catalyst in DMF, under microwave irradiation at 80 °C for 7 h, and under inert atmosphere [78].

(B) Sonochemistry-based synthesis of coumarins.

Substituted coumarins were prepared via sonochemistry using active methylene compounds and 2-hydroxybenzaldehydes (Knoevenagel reaction) or resorcinol (Pechmann reaction). Good yields, short reaction times and easy adaptability to bulk production are sure advantages of this procedure [79].

(C) Synthesis of azidocoumarins for click reactions.

**Scheme 7:**
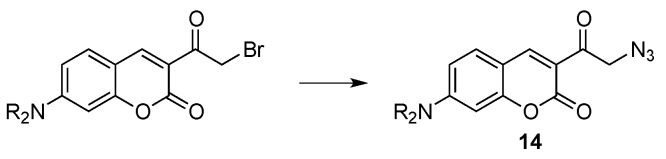
Synthesis of 3-azidoacyl coumarins from 3-bromoacyl precursors.

**Scheme 8:**
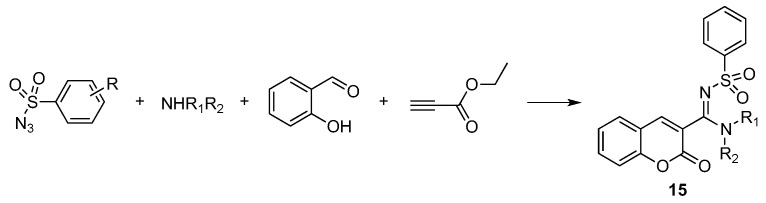
Four-component synthesis of 3-sulphonylamidine coumarins.

Many azidocoumarins have been prepared [80,81] as fluorescent reagents for the synthesis of substituted 1,2,3-triazoles through the 1,3-dipolar click cycloaddition. Evans and coll. [80] have reported the preparation of a series of 7-*N*-alkyl and *N*,*N*-dialkylamino coumarins **14** (Scheme 7) bearing an azidoacyl group at position 3, starting from the corresponding 3-acylcoumarins.

(D) Synthesis of coumarins by multicomponent reactions

  (D1) Synthesis of 3-*N*-sulphonylamidine coumarins **15** (Scheme 8).

**Scheme 9:**
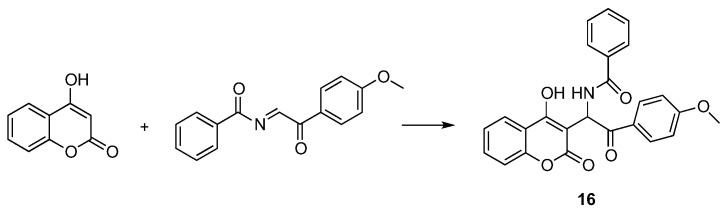
Multicomponent synthesis of 3-aroylamido coumarins.

The synthesis of title coumarins has been accomplished with moderate to high yields, by the coupling of salicylaldehydes, propiolates, sulfonyl azides, and secondary amines [82]. The four-component tandem reaction was carried out in 1,4-dioxane, in a sealed tube and inert atmosphere, at 130 °C, under microwave irradiation, by using copper(I) iodide as catalyst.

  (D2) Synthesis of 3-aroylamido coumarins **16** (Scheme 9).

The title coumarins were prepared in high yield via an efficient, one-pot, three-component reaction of aryl glyoxals, benzamides, and 4-hydroxycoumarins in the presence of molybdate sulfuric acid under solvent-free conditions at 80 °C [83].

It is worth noting that although coumarins can be obtained either from diverse plant sources, or, as just seen, from a variety of chemical reactions, bulk production of diverse coumarin derivatives can be better realized through suitably oriented biosynthetic pathways by using microorganisms such as bacteria (e.g., *Escherichia coli*) [84,85], and fungi, that is *Basidiomycetes* and *Ascomycetes* [86].

## 3. Coumarins as Enzyme Inhibitors

Bio-pharmacological activity of coumarins on a limited number of targets will be fully analyzed. In particular, the bio-pharmacological activity of 3, 4, and 7-monosubstituted coumarins and 3,4,7-polysubstituted coumarins will be mostly taken into account.

### 3.1. Coumarins as MAO Inhibitors

MAOs are the enzymes responsible for the oxidative deamination of exogenous and endogenous amines, including neurotransmitters. MAOs exist as two isoforms, MAO A and MAO B, differing from selectivity towards substrates and sensitivity to inhibitors [87]. Selective MAO A inhibitors are currently used as antidepressants, while MAO B inhibitors are used in combination with levodopa in the treatment of Parkinson’s disease [88].

**Figure 7:**
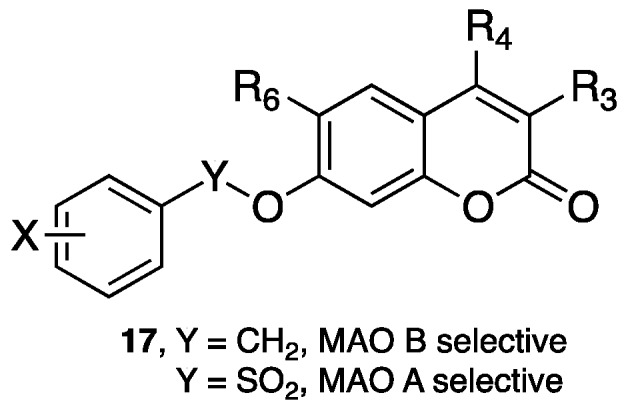
7-Benzyloxy and 7-arylsulfonyloxycoumarins as monoamine oxidase (MAO) inhibitors.

The first description of synthetic coumarins as MAO inhibitors dates back to 1994, when a joint publication from BASF and Knoll AG described the inhibitory activity of two previously patented series of 7-arylalkoxycoumarins [89] and 7-arylsulfonyloxycoumarins [90] as highly selective MAO B and MAO A inhibitors, respectively [91]. Such outstanding activity and selectivity were further investigated by the teams of Carotti and Testa, who prepared a larger series of 7-benzyloxy and 7-arylsulfonyloxycoumarins of the general formula **17** illustrated in Figure 7. The X-substituents were selected in order to deeply explore their physicochemical domains in terms of electronic, lipophilic, and steric properties [92]. The main structural requirements for high MAO A and MAO B affinity and B/A selectivity were suggested. Potent and selective inhibitors with IC_50_s in the nanomolar range were found. The inversion of selectivity from 7-benzyloxy derivatives (B-selective) to 7-sulfonyloxy esters (A-selective) was interpreted by CoMFA analyses in terms of different spatial distribution of electron density and lipophilicity. The CoMFA isocontour maps helped at the 3D level in terms of the interpretation of SARs. For MAO B selective inhibitors, a close relationship between potency and lipophilicity was shown for *meta* substituted benzyloxy derivatives, with the derivation of the following regression Equation (1):

pIC_50_ (MAO B) = 0.19 ± 0.15 π − 0.56 ± 0.21 π^2^ + 8.46 ± 0.14 (*n* = 11; *r*^2^ = 0.86) (1)

The dependence of selectivity from the chemical nature of the bridge linking the aromatic substituent to the coumarin core was further investigated through ligand- and target-based approaches [93]. Most of newly synthesized derivatives displayed MAO B inhibitory activity (as IC_50_s) in the nanomolar range. Docking studies on rat MAOs (homology model) showed that the oxymethylene linker of 7-benzyloxy derivatives fits the entrance cavity of MAO B, and may establish a hydrogen bond between oxygen and Tyr326 surrounding the entrance cavity of the enzyme. Differently from MAO B, MAO A entrance cavity may accommodate bulkier groups, and shows great affinity for the 7-sulfonyloxy linker, which acts as a H-bond acceptor. For both MAO A and B affinities, the presence of hydrophobic amino acids caging the entrance cavity favors the hydrophobic interaction of aryl substituents in position 7. Moreover, substitution of positions 3 and 4 of the coumarin core with methyl or other small groups (e.g., cyano, chlorine) increased the affinity for MAO B of about one order of magnitude. Evaluation of the results from docking and QSAR models drove the design and synthesis of a new congener, that is the 3-methyl-7-(3-chlorobenzyloxy)coumarin, displaying outstanding MAO B affinity (IC_50_ of 5.1 nM) and B/A selectivity index (SI = 2500).

The opportunity of shifting from rat to human enzymes, prepared through recombinant biotechnologies and made commercially available, urged the search for possible correlations between activities on human enzymes and those on rat enzymes. Inhibitory activity on rat MAOs (rMAOs) are assessed directly on homogenates of fresh brain tissues, and might be significantly influenced by microenvironmental features of the tissues, and possible tissue contamination, which is very difficult to quantify. As a consequence, data from rat brain homogenates and cloned enzymes are not comparable, being influenced by different, and not quantifiable, factors. By contrast, activities determined on highly pure recombinant human MAO B (hMAO B) with a set of known MAO B selective inhibitors, were comparable with values on hMAO B from blood platelets, a “cleaner” source of MAO enzymes [94].

A screening of a larger number of inhibitors on hMAO B led to IC_50_s about 10-fold lower than those determined for the rat isoform [95]. A similar comparison of rat and human MAO A indicated an even more pronounced increase of affinity going from rat to human isozymes, generally leading to a diminution of B/A selectivities. However, no clear quantitative relationships were found between inhibition data from MAO of different species, in particular, between rat and human MAOs.

**Figure 8:**
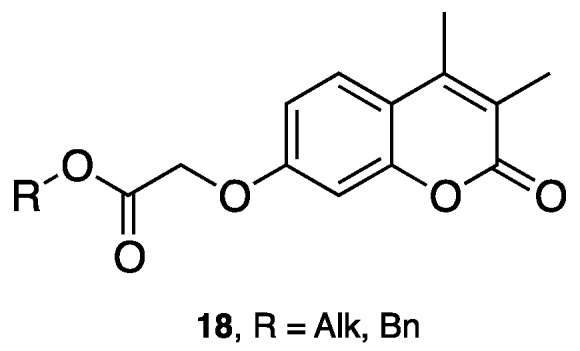
Ester derivatives of 7-hydroxy-3,4-dimethylcoumarin as rMAO inhibitors.

The dependence of MAO affinity, particularly of MAO B, from lipophilicity, was confirmed by a QSAR study on a new series of 7-substituted coumarins bearing ether, ester, and sulfoxide groups as linkers [96]. rMAO B affinities resulted in a good linear correlation with logP (Equation (2)), while in subseries bearing the ester group as the bridging function (**18**, Figure 8), the steric hindrance of R correlated with the hydrolytic half-life of esters in buffer. For the first time on this class of coumarins, an ex vivo study on rat liver and brain was performed: favorable brain permeation was demonstrated, but a drop of selectivity was found ex vivo, compared to in vitro data.

pIC_50_ (MAO-B) = 1.18 ± 0.17 log*P* + 3.26 ± 0.56 (*n* = 20; *r*^2^ = 0.716) (2)

**Figure 9:**
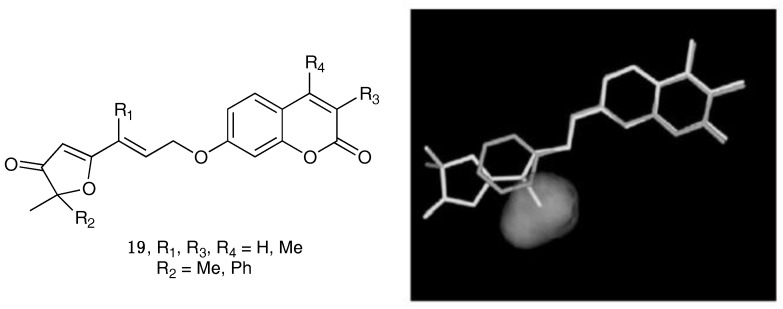
Left, chemical structure of geiparvarins **19** [98]. Right, molecular overlay of 7-benzyloxy-3,4-dimethylcoumarin (grey) onto 3,4-dimethylgeiparvarin (white). The van der Waals surface of the 3′-methyl group impacts the region of the *ortho*-substituents in the phenyl ring of 7-benzyloxycoumarins. Reproduced with permission of Elsevier.

The MAO inhibition study was then extended to a class of natural compounds, the geiparvarins, which are 7-substituted coumarins extracted from *Geijera parviflora* leaves. They had been investigated as antitumor agents for their cytostatic properties [97], but their close similarity with 7-arylalkoxycoumarins suggested a potential activity as MAO B inhibitors. Biochemical assays of a series of natural and newly synthesized geiparvarins **19**, illustrated in Figure 9, confirmed a good rMAO B inhibitory activity in the low- to submicromolar range, and a strong MAO B/A selectivity [98]. Structural modifications on either the coumarin or the furanone moiety of geiparvarin are mostly deleterious for MAO activity. By contrast, removal of the methyl group on the alkenoxy bridge afforded a derivative (R_1_, R_3_, R_4_ = H and R_2_ = Me) which displayed the highest MAO B inhibitory potency (IC_50_ = 28 nM) with a high B/A selectivity (850-fold). The lower affinity of geiparvarin derivatives bearing a methyl group at position R_1_ was interpreted on the basis of a similarly decreased activity displayed by *ortho*-substituted-7-benzyloxycoumarin derivatives. In fact, while the X-ray structure of 3,4-dimethylgeiparvarin can well overlap the molecular skeleton of the potent and selective MAO-B inhibitor 3,4-dimethyl-7-benzyloxycoumarin, as shown in Figure 9, the 3′-methyl analogue places the methyl group into the same unfavorable region of the *ortho*-substituted 7-benzyloxy derivatives.

**Figure 10:**
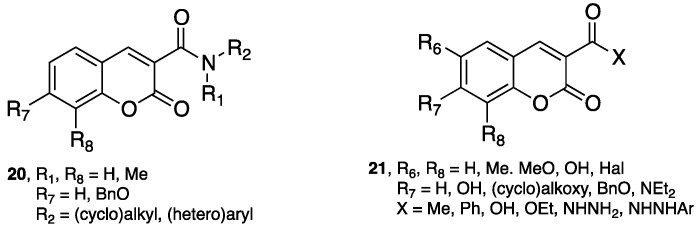
3-Carboxamidocoumarins **20** and 3-acylcoumarin derivatives **21** as hMAO inhibitors.

The easy synthetic functionalization of coumarin scaffold allows the exploration of different patterns of substitution. The introduction of aryl and heteroaryl groups in position 3, either tethered by a simple chemical function, or directly linked to the coumarin core, resulted in potent hMAO inhibitors with different B/A selectivity according to the presence of additional substituents on the coumarin core, and/or on the aryl in position 3. Chimenti and coll. [99] studied the effects of substitution on the phenyl substituent of 3-carboxanilidocoumarins **20** (Figure 10), tested on hMAOs with IC_50_s spanning from micro- to submicromolar range.

**Figure 11:**
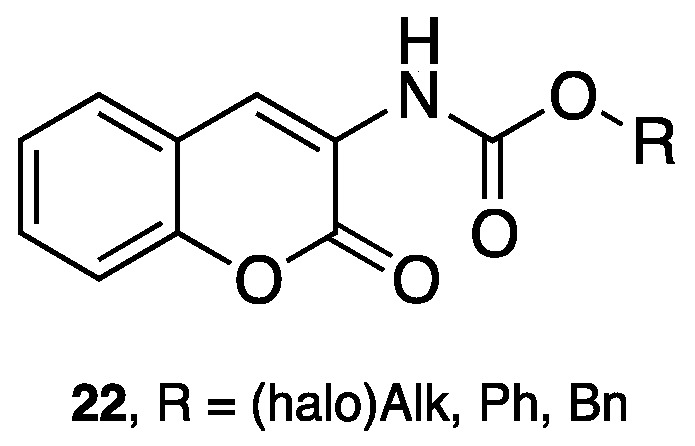
3-Carbamylcoumarins **22** as MAO inhibitors.

Docking studies outlined a good accommodation of even bulky substituents in position 3, leading to 4-(methanesulfonyl)anilido derivative as the most potent MAO B inhibitor (IC_50_ = 1.4 nM). However, the introduction of benzyloxy in position 7 led to a drop of MAO B affinity. The same research group extended this approach to different 3-carbonyl derivatives **21** (Figure 10), including ester, acyl, and hydrazido derivatives, variously substituted at the 5, 6, 7, and 8 positions of coumarin. Indeed, this led to inhibitors with strongly decreased potencies, with the notable exception of 7-benzyloxy-3-ethylester derivatives, and of 3-carboxyhydrazidocoumarin, showing strong hMAO B inhibition (IC_50_ = 3.2 nM) and high hydrolytic stability [100]. A systematic variation on this structural motif led to carbamate **22** (Figure 11) [101], still showing high B/A selectivity, but with lower inhibitory potencies compared with 7-benzyloxycoumarins analogues previously described.

**Figure 12:**
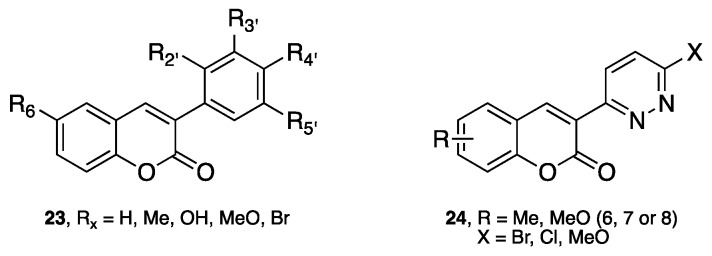
3-Phenyl- **23** and 3-pyridazinylcoumarins **24** as hMAO inhibitors.

A strong improvement in both hMAO B inhibitory potency and selectivity was achieved by the same group, by preparing a series of 3-phenyl-6-substituted coumarins **23** (Figure 12) with IC_50_s in the submicro- to subnanomolar range [102]. The presence of a substituent in the less explored position 6 of coumarin appeared well tolerated, unless a bulky group was inserted. A subsequent refinement of this structural motif, with the introduction of small substituents (i.e., methyl, methoxy, bromine) in the 3-phenyl ring, led to potent and selective hMAO B inhibitors, hitting an IC_50_ of 134 pM for 6-methyl-3-(3-bromophenyl)coumarin [103]. An extension of SAR within the class of 3-phenylcoumarins, with the introduction of a hydroxyl in position 4, resulted in less effective inhibitors, probably because of a steric clash with Tyr326, as suggested by docking studies [104]. Finally, the isosteric replacement of 3-phenyl ring with pyridazine returned good B/A selectivity and favorable predicted absorption, distribution, metabolism, excretion, and toxicity (ADMET) properties, although hMAO B affinities were in the micromolar range (**24**, Figure 12) [105].

The position 4 of coumarin nucleus is also a well-investigated stem of molecular diversity, for the modulation of MAO affinity, selectivity and, particularly important, the pharmacokinetic properties. In most cases, 4-substituted coumarins, possibly bearing additional substituents on the positions 6 and/or 7, retain potential in MAO B binding.

**Figure 13:**
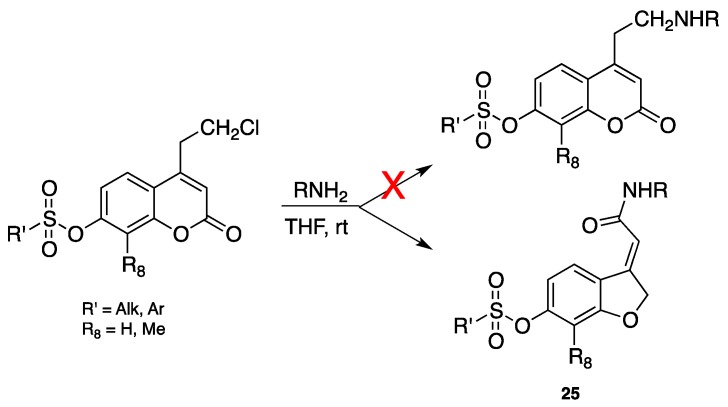
Benzofuran derivatives **25**, obtained from the opening and rearrangement of coumarin lactone ring, as detailed in Ref. 106.

Incidentally, while attempting to improve the pharmacokinetic properties of the 7-arylsulphonyloxy coumarins by introducing polar amino moieties at position 4 (Figure 13), an unexpected side reaction took place. In the reaction with secondary aliphatic amines, 4-(chloromethyl)coumarin 7-sulfonate esters underwent an opening and rearrangement of the lactone ring, affording 3-substituted benzofuran 6-sulfonate esters (**25**, Figure 13), which fully retained potency and selectivity for rMAO A over rMAO B [106].

**Figure 14:**
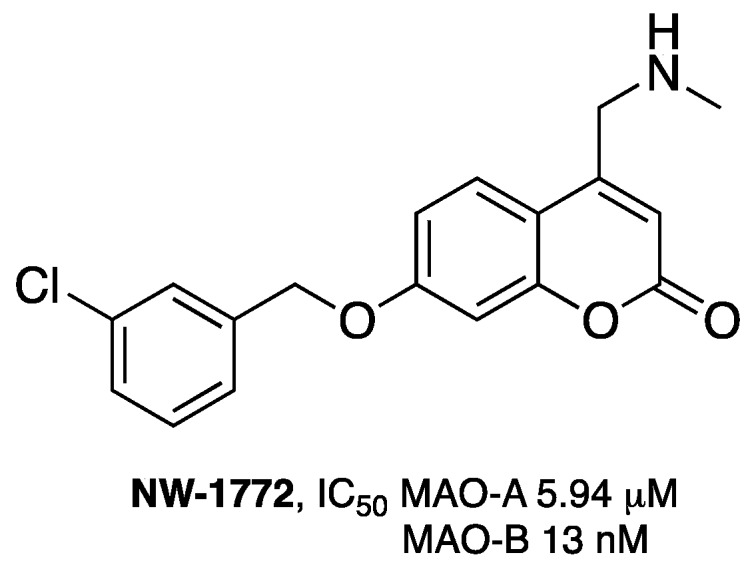
Structure and rMAO inhibitory activity of **NW-1772**.

Among the class of 4-substituted coumarins, 7-[(3-chlorobenzyl)oxy]-4-[(methylamino)methyl]coumarin (**NW-1772**) [107,108,109] (Figure 14) was found as a hit compound for its high rMAO B inhibitory potency, B/A selectivity, and interesting pharmacokinetic properties. This compound was selected among a wide variety of 7-*meta*-chlorobenzyloxy coumarin derivatives bearing polar substituents at position 4 (amine, amide, ether, ester, alcohol functions). Compound **NW-1772** showed good blood–brain barrier (BBB) permeability, low cytotoxicity, and impressive MAO B/A selectivity in the ex vivo rat brain MAO functionality assay. A linear dose–response plot resulted for a wide range of administered doses. **NW-1772** was patented by Newron and some of us, along with a series of close derivatives [107,108].

**Figure 15:**
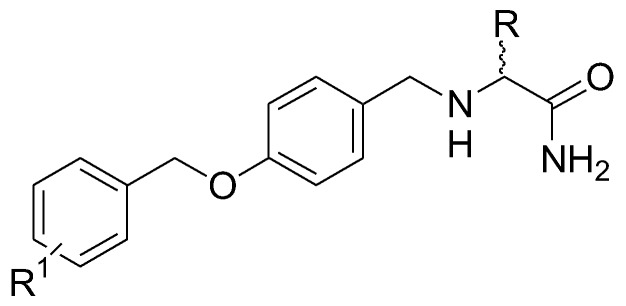
General structure of safinamide analogues with rMAO B inhibitory activity.

Open analogues of the coumarin **NW-1772** were prepared through a solid phase synthesis, leading to safinamide and a library of alkylamidic congeners (Figure 15). Safinamide, that is (*S*)-*N*_2_-(4-((3-fluorobenzyl)oxy]benzyl)alaninamide methanesulfonate, recently entered the clinic with the name of Xadago^®^ as add-on to levodopa for the treatment of mid- to late-stage Parkinson’s disease [110]. Safinamide and its analogues were tested for their rMAO inhibitory activity and selectivity. Interestingly, two derivatives, (*S*)-3-chlorobenzyloxyalaninamide and (*S*)-3-chlorobenzyloxyserinamide, resulted in more potent MAO B inhibitors than safinamide (IC_50_ 33 and 43 nM, respectively, vs. 98 nM) but with a lower MAO B selectivity index (SI 3455 and 1967, respectively, vs. 5918) [111].

**Figure 16:**
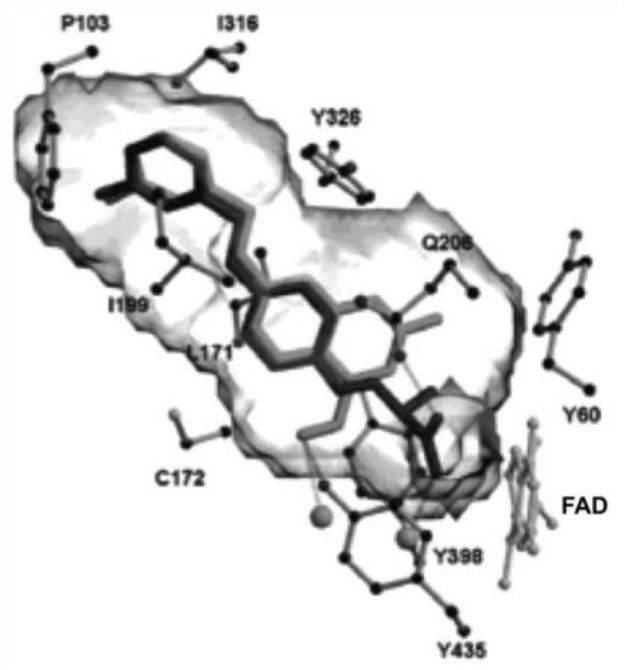
Overlay of safinamide (black) and 7-(3-chlorobenzyloxy)-4-carboxaldehyde-coumarin (gray), a strict analogue of **NW-1772**, onto the hMAO B active site [112]. The active site residues and the FAD cofactor are also shown. Reproduced with permission of ACS.

A major breakthrough in the design of MAO inhibitors stemmed from the X-ray resolution of co-crystallized complexes of hMAOs with a series of inhibitors, either reversible or irreversible. Co-crystallization of **NW-1772**, and close analogues, with hMAO B [112] and safinamide (Figure 16) outlined the main molecular interactions into the active site of the enzyme, and confirmed most of the observations derived from docking simulations on in silico homology models, particularly the position of coumarin ring in front of the FAD cofactor, and of the benzyloxy residue spanning over the entrance cavity lined by a series of side chains of hydrophobic amino acids, including the “gate-keepers”.

Keeping in mind the informative structural data coming from X-ray studies, a structure-based design of a more extended series of selective MAO B inhibitors [113], was made by keeping a 7-*meta*-chlorobenzyloxy substituent, to assure favorable and selective binding to rMAO B, and introducing polar or even charged substituents in position 4. Very satisfactorily, MAO B affinity and B/A selectivity were well retained in most of the designed compounds, which in addition, showed lower lipophilicity and higher water solubility, two good prerequisites for a progression towards pre-clinical and clinical studies. For this purpose, the design and synthesis of effective pro-drugs or “soft” drugs with favorable ADMET properties, was successfully investigated by introducing metabolically sensitive functions at position 4.

**Figure 17:**
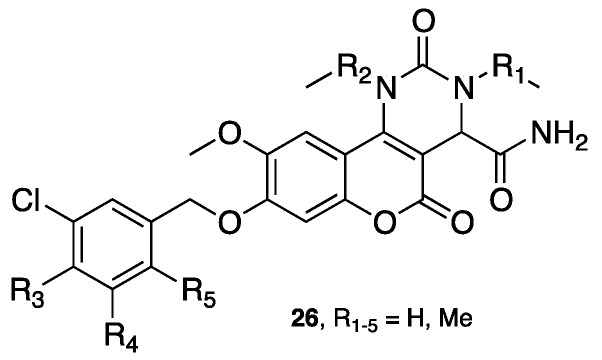
Computer-assisted design of coumarin derivatives as potent hMAO B inhibitors.

**Figure 18:**
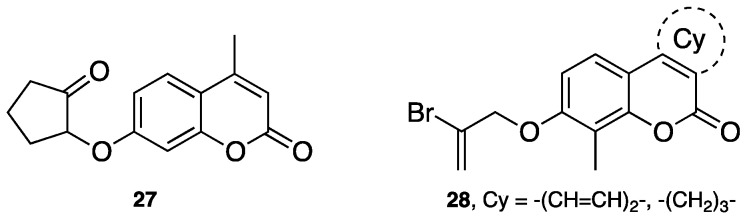
Computer-assisted design of selective hMAO A/B inhibitors.

Taken together, the findings described above paved the way to a robust target-based design of coumarin ligands, moving beyond the less reliable docking on homology models and the ligand-based inhibitor design. Indeed, the X-ray structures of hMAO B-inhibitor complexes [112] have been used by many researchers, reaching up now more than 160 citations in Scopus. Since the appearance of that seminal paper, several computational studies (computer-assisted molecular design) have been performed to identify and rationalize the most important molecular determinants for a high MAO affinity and MAO A/B selectivity, and to confer drug-likeness features to newly designed inhibitors. Very recently, by applying both ligand- and target-based screening campaigns of known, coumarin-based selective MAO inhibitors, Mladenovic and coll. [114] derived new models that drove the synthesis of new 7-benzyloxycoumarin derivatives with nano- to subnanomolar activity on hMAO B, and outstanding B/A selectivity (**26**, Figure 17). By a similar approach, Santana and coll. had previously developed and successfully applied a computational method (MARCH-INSIDE) [115] and a combined QSAR-CN (complex networks) model [116] to the preparation of highly selective hMAO A and B inhibitors (**27** and **28** respectively; Figure 18) bearing coumarin as the core scaffold. More recently, a molecular dynamics (MD) study investigated the structural elements guiding hMAO selectivity of 7-substituted coumarins [117]. For the first time, MD simulations have been applied to the study, at a molecular level, of the interaction of selective coumarin inhibitors with hMAO A and hMAO B. A major outcome was the detection of a water-mediated hydrogen bond between the MAO B selective 7-benzyloxycoumarin and FAD, an interaction quite different from that formerly predicted by docking of close analogues [93].

### 3.2. Coumarins as ChE Inhibitors

Acetyl- and butyrylcholinesterase (AChE and BChE), particularly AChE, are responsible for the catalytic degradation of neurotransmitter acetylcholine (ACh) into the synaptic cleft of cholinergic nerves. At the CNS level, their inhibition restores the levels of ACh in the neuronal systems damaged by neurodegeneration. From this observation stemmed the so-called cholinergic hypothesis for the interpretation of the onset and progress of AD [118]. The use of AChE inhibitors in the therapy of Alzheimer’s disease (AD) has since then ensured a continuous interest in the search for new molecules acting as AChE/BChE inhibitors. Coumarin derivatives, either from synthetic and natural origin, are a well-pursued class of molecules acting as AChE/BChE inhibitors, so that many reviews have been devoted to this topic, even very recently [11]. A very “trendy” approach to the design of ChE inhibitors led to conjugated molecules containing the coumarin moiety linked to a known inhibitor of ChEs, typically donepezil or tacrine, in order to get the highest inhibition of the enzyme. Although no co-crystallized complex of such coumarin hybrids with human ChEs (hChEs) is available yet, docking studies and inhibition kinetics suggest for these molecules a typical binding pose stretching from the catalytic (CAS) to the peripheral active site (PAS) of the enzyme, whence the definition of these compounds as dual binding site (DBS) inhibitors. A confirmation of the preferred binding of the coumarin ring into the peripheral site has been obtained from the crystal structure of the complex of *Torpedo californica* AChE (TcAChE) with aflatoxin [119].

Many of such coumarin derivatives have been described as DBS inhibitors of AChE, with different potencies according to the nature and strength of the interactions involved. Usually, the coumarin ring is accommodated into the peripheral binding site, due to its planarity, steric complementarity, and possible π–π interactions with the aromatic residues of the enzyme herein located, while, in general, a net positive charge (e.g., ammonium salts), or a protonatable nitrogen, allows strong cation–π interactions within the catalytic site.

**Figure 19:**
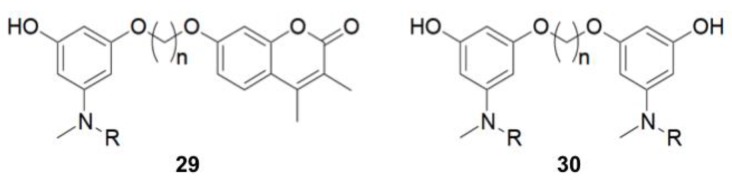
General formulae of DBS bAChE inhibitors.

In 2004, our group published the synthesis of DBS bovine (bAChE) inhibitors, realized through solid phase techniques [120]. The synthetic protocol consisted of three subsequent Mitsunobu reactions: first of all, the coupling of dimethylaminophenol with the brominated Wang resin was performed with DIAD and PPh_3_ in THF, while the two consecutive Mitsunobu reactions, consisting in the introduction of an aliphatic spacer bringing a second phenolic moiety, were performed in presence of ADDP and PBu_3_ in CH_2_Cl_2_. By this synthetic way, amines with the general structures **29** and **30** (Figure 19) were prepared.

**Figure 20:**
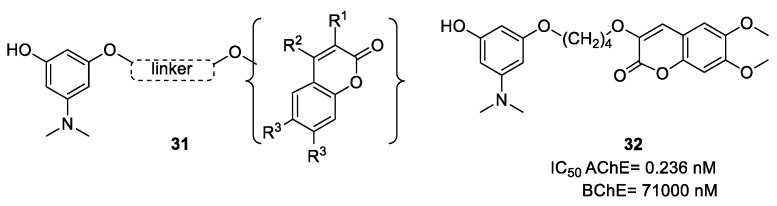
bAChE-selective coumarin inhibitors.

Cation–π and π–π interactions were suggested for coumarin–edrophonium heterodimers **29**, where a 3,4-dimethyl-7-hydroxycoumarin fragment was tethered to an edrophonium-like moiety [121]. While the neutral amino analogs had only a fair AChE affinity, ammonium salts reached low nanomolar IC_50_ values. All of them showed high bAChE over equine serum (esBChE) selectivity. As a prosecution of this work, in 2010 our group published the synthesis and the biological evaluation as bAChE and esBChE inhibitors of a large series of substituted coumarins linked through an appropriate spacer to 3-hydroxy-*N*,*N*-dimethylanilino or 3-hydroxy-*N*,*N*,*N*-trialkylbenzaminium moieties. The best bAChE inhibitory activity in this series (**31**) was obtained with the 6,7-dimethoxy-3-substituted coumarin derivative **32** (Figure 20, IC_50_ = 0.236 nM), that also showed high AChE/BChE selectivity (SI > 300,000). The AChE affinity of many 3-hydroxy-*N*,*N*,*N*-trialkylbenzaminium salts was in the sub-nanomolar to picomolar range, and their AChE/BChE selectivities were also impressive (SI values up to 138,000). Docking studies and molecular dynamics (MD) simulations allowed for the detection of two possible alternative binding modes, and confirmed the important role of π–π stacking interactions with the hAChE PAS [122].

**Figure 21:**
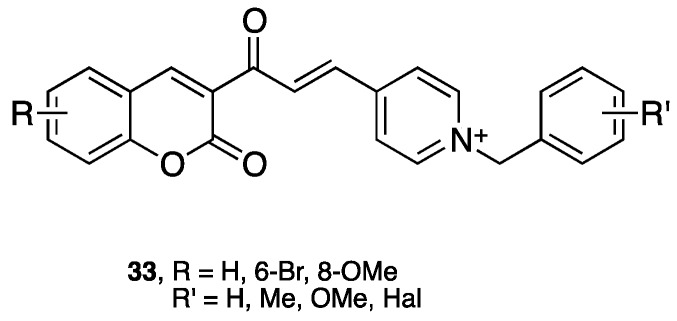
Coumarin–pyridinium derivatives as ChEs inhibitors.

A similar approach was later used in the design of a series of coumarin–pyridinium congeners [123], when the pyridine nitrogen was transformed into quaternary benzylammonium salt, leading to potent electric eel (eeAChE) inhibitors with IC_50_s in the nano- to picomolar range (**33**, Figure 21). Also in this class of inhibitors, the substitution of coumarin was in position 3, to warrant an efficient binding of the coumarin moiety into the PAS.

**Figure 22:**
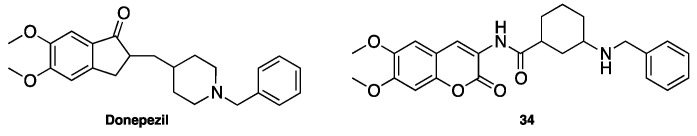
Donepezil-like coumarin **34** as bAChE selective inhibitor.

In 2013, our group published the design, preparation, and biological assays of a series of coumarin alkylamines, presenting some structural features of donepezil by combining target- and ligand-based approach. Donepezil (Figure 22) is a DBS reversible inhibitor of AChE, very selective over BChE, and is one of most used AChE inhibitors in AD therapy. Some 6,7-dimethoxycoumarin derivatives with a protonatable benzylamino group, linked to position 3 by diverse linkers, presented good bAChE inhibitory activity and selectivity over esBChE. The length and shape of the linker and the presence of methoxy substituents on the coumarin scaffold were determinant for the inhibitory potency. The most active compound **34** (IC_50_ 7.6 nM, Figure 22) showed a mixed-type inhibition mechanism, confirming the binding at both the CAS and PAS of bAChE [124].

**Figure 23:**
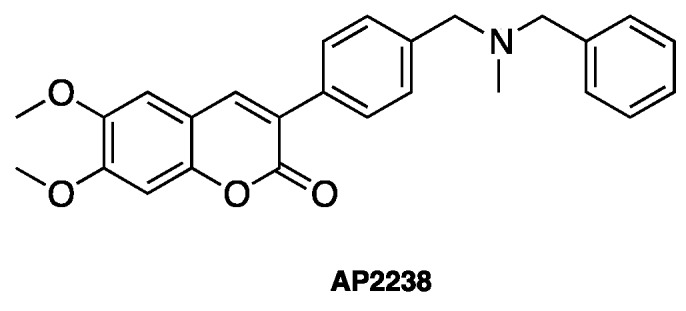
Chemical structure of hAChE selective inhibitor **AP2238**.

**Figure 24:**
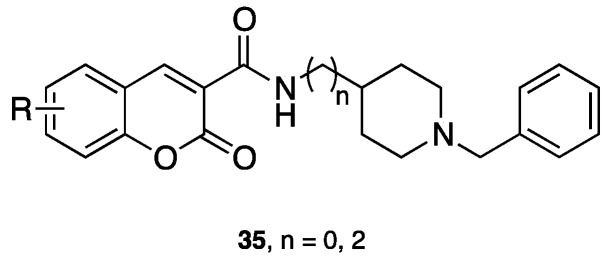
3-Carboxamidocoumarins **35** as eeAChE selective inhibitors.

**Figure 25:**
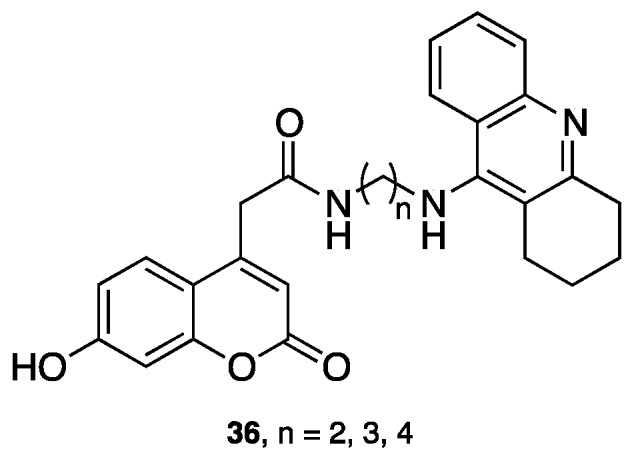
Example of tacrine–coumarin hybrids **36** as potent hChEs inhibitors.

One of the first descriptions of donepezil-like compounds was released in early 2000s by Piazzi and coll., who reported very good hAChE inhibitory activities in a class of 3-benzylaminocoumarins, among which **AP2238** (Figure 23), bearing the 6,7-dimethoxycoumarin, was progressed to further biochemical and pharmacological tests [125]. On the same wave, other authors described donepezil–coumarin hybrids **35** (Figure 24), bearing small substituents in positions 6, 7, or 8 of the coumarin scaffold, with nanomolar affinities on eeAChE, high AChE/BChE selectivity, and promising neuroprotection against oxidative stress [126]. The hybridization of tacrine–coumarin is also a recurrent approach for the design of potent ChE inhibitors, as for example, derivative **36** (Figure 25) [127].

### 3.3. Coumarins as Multitarget MAO–ChE Inhibitors

**Figure 26:**
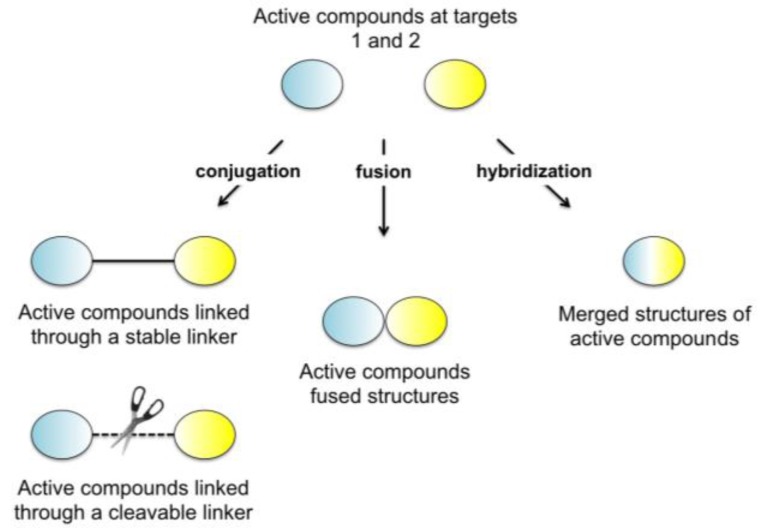
Multitarget drug design strategies.

The multitarget approach to address neurodegenerative and other complex diseases has become a “new paradigm” in the research for new and more efficient drugs [128]. Due to their multifactorial etiology, the therapeutic protocol for multifactorial diseases normally involves the administration of a drug cocktail, with increased risks of drug–drug interactions and toxicity. The concept “one drug, multiple targets”, underlying the multitarget approach, aims to confer to a single drug, different pharmacological properties amenable to the same disease therapy [129]. The advantages of such monotherapy lie mostly in the lack of drug–drug interactions, in a higher compliance of patients assuming only one drug for their disease, and easier ADMET profiling and pharmacological and therapeutic characterization. On the other hand, such multitarget profile needs an accurate balancing of the potencies, in order to ensure the desired pharmacological effects on each of the targets addressed. The simpler, yet straightforward way to prepare multitarget ligands (Figure 26) consists in attaching two pharmacophoric moieties, responsible for two diverse bio-pharmacological activities, by means of a stable or metabolically cleavable linker (conjugation), or by a direct covalent bond formation (fusion). A more sophisticated multitarget ligand design [130,131] consists in the merging (hybridization) of two or more pharmacophoric moieties in a new unique molecular entity, able to display the original activities of both moieties. A caveat in designing fused/conjugated molecules is that it often results in very large molecules with high molecular weight, high number of rotatable bonds, high lipophilicity, and low aqueous solubility. Consequently, these molecules would likely display poor pharmacokinetic properties, particularly low bioavailability and high propensity to become substrates for detoxification systems. Hybrid molecules, in contrast, may retain higher druglikeness, and could undergo an easier development as hit compounds for pharmacological studies.

Because of the well-known interactions of coumarin nucleus within the binding sites of MAOs and ChEs, molecular conjugates containing this scaffold have been widely described in literature as dual ChE–MAO inhibitors. A seminal, pioneering paper issued by the groups of Testa and Carotti in 2001 reported the eeAChE inhibition displayed by 7-benzyloxycoumarins **17** (Figure 7) previously described as rMAO B selective inhibitors [132]. All acted as noncompetitive/mixed inhibitors of eeAChE in a 3–100 μM range. Building on this evidence, new coumarin derivatives featuring different patterns of substitution were described later, by the same and other groups.

**Figure 27:**
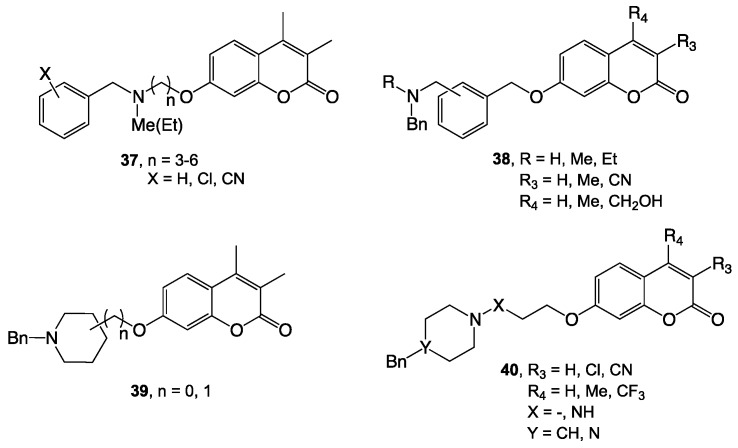
7-Substituted coumarins bearing protonatable groups for dual ChE–MAO inhibition.

In a first step, the introduction of a benzylamine moiety on the coumarin skeleton with a nitrogen protonatable at physiological pH was explored. The designed compounds augmented eeAChE inhibition and retained good rMAO B affinity, thus leading to AChE–MAO B dual inhibitors with unprecedented potencies in the nanomolar range for hMAO B and submicromolar for hAChE [133]. The basic *N*-benzyl group was linked to the position 7 by means of a flexible alkoxy chain (**37**), or a more rigid 7-benzyloxy moiety (**38**, Figure 27). An important structural modification was performed with the introduction of a hydroxymethyl group in position 4 of the coumarin, leading to inhibitors with improved aqueous solubility and unchanged MAO B potency and selectivity. The good balance of AChE and MAO B activities and the high MAO B/A selectivity, along with a preliminary and positive assessment of ADMET properties, made this class of congeners promising hit structures for further development.

The same structural feature was retained while replacing the 7-benzyloxy substituent with a *N*-benzylpiperidine, the pharmacophoric moiety of donepezil. In this new series of compounds, the effects of topology of piperidine substitution (**39**, Figure 27) were also investigated. Although the 3-substituted piperidine derivatives scored better results than the 4-substituted regioisomers as rMAO B inhibitors, overall, this strategy led to multitarget AChE–MAO B inhibitors with high potency and selectivity, low cytotoxicity, high cytoprotection from oxidative insults, and very favorable pharmacokinetic profiles, particularly good aqueous solubility and BBB permeability [134]. A subsequent study confirmed this excellent activity profile for highly close congeners **40** (Figure 27) [135].

**Figure 28:**
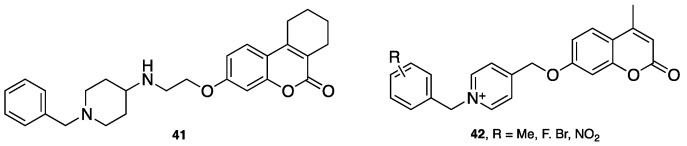
7-Substituted coumarins as dual ChE/MAO inhibitors.

**Figure 29:**
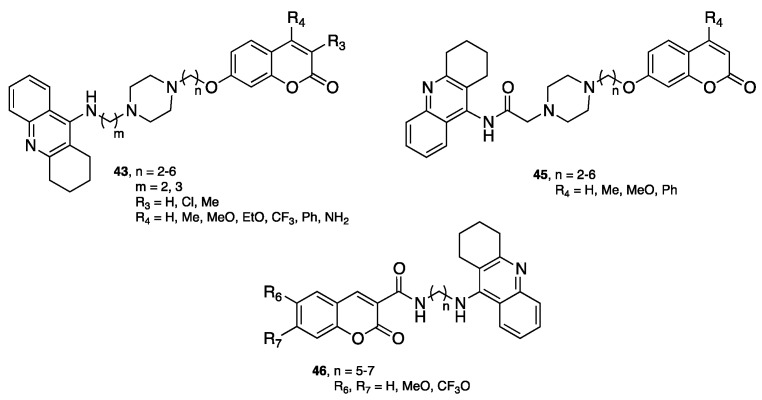
Coumarin–tacrine conjugates as multitarget-directed ligands.

The introduction of a second basic nitrogen in the linker, as in compound **41** (Figure 28) [136], as well as the replacement of benzene ring of the benzyloxy moiety with a charged *N*-benzylpyridinium group [137] (**42**, Figure 28) led to molecules with retained multitarget activity, but with decreased potency. Particularly, hMAO B affinity was negatively affected by the introduction of charged residues. As expected, better activity profiles were found by the same authors for coumarin–tacrine conjugates **43** (Figure 29), where the length of the spacer allowed a more pronounced increase of hBChE and hMAO B affinity, despite the presence of a protonatable piperazine linker [138].

**Figure 30:**
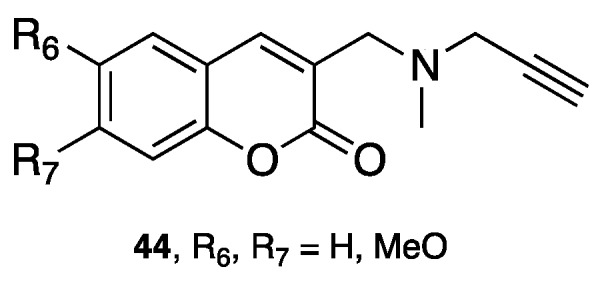
3-Propargylamine derivatives of coumarin as dual ChE–MAO inhibitors.

The topological shift of the basic moiety to the position 3 of the coumarin ring was detrimental for rMAO activity, with the valuable exception of *N*-propargylamine derivatives **44** (Figure 30), which of course may act with a different mechanism of MAO B inhibition. Such structural features, present in a known class of MAO irreversible inhibitors in therapeutic use (i.e., selegiline and rasagiline) led to the retention of a fair dual AChE–MAO B inhibitory activity, with good BBB permeability and cytoprotective effects [139].

### 3.4. Coumarins as Pleiotropic Agents in NDs

The multitarget approach for the treatment of NDs, and other multifactorial diseases [140], aims to confer multiple activities to a single molecular entity, all amenable to a synergistic therapeutic effect. Even if the design of such pleiotropic entities suffers from an evident complexity in properly addressing, combining, and balancing all the desired pharmacological activities in a single multitarget molecule [141], the therapeutic potential of such molecules towards different and important diseases could be significantly improved.

**Figure 31:**
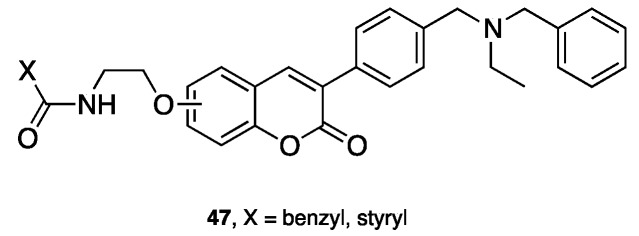
3-Phenylcoumarins as multitarget-directed ligands.

The easy synthetic access to a wide number of coumarin derivatives makes the coumarin scaffold an amenable structural tool for the preparation of molecular hybrids [142]. A simple, yet effective, strategy in designing new pleiotropic agents moves from a molecule with known biological activity, by decorating and/or conjugating it with appropriate moieties, and trying to retain the original activity while looking for the wanted additional one(s). By this strategy, Xie and coll. [143] described anti-amyloid and metal chelating properties in a series of 7-hydroxycoumarin–tacrine conjugates **45** (Figure 29), prepared to act as efficient inhibitors of eeAChE/esBChE. The two moieties were conjugated through a spacer, bearing a piperazine ring linked by an amide bond to tacrine. Authors indicated this amide bond as the chelating moiety for copper(II), but no convincing rationale and experimental data were provided. ChE inhibitory potencies were congruent with those described by authors in other publications [138], and fair inhibition of self-induced aggregation of beta-amyloid 1-42 (Aβ42) was also disclosed. Similarly, 3-carboxycoumarin–tacrine conjugates **46** (Figure 29) showed potent, yet non-selective, hAChE/BChE inhibition with additional β-secretase (BACE1) and Aβ aggregation inhibitory activities [144]. The possibility to address BACE1, one the most pursued targets in the search for anti-amyloid agents, drove the attention of many researchers to multitarget agents endowed with BACE1 inhibitory activity. As for dual AChE–BACE1 inhibition, this activity profile was found for the first time in a series of substituted 3-phenylcoumarins, where the balance of potencies favored the BACE-1 inhibition, with IC_50_s in the nM range [145]. The design rationale moved from AChE inhibitor **AP2238** [125] (Figure 23), and involved its decoration with an arylacetamide linked to the coumarin ring through a short spacer (**47**, Figure 31).

**Figure 32:**
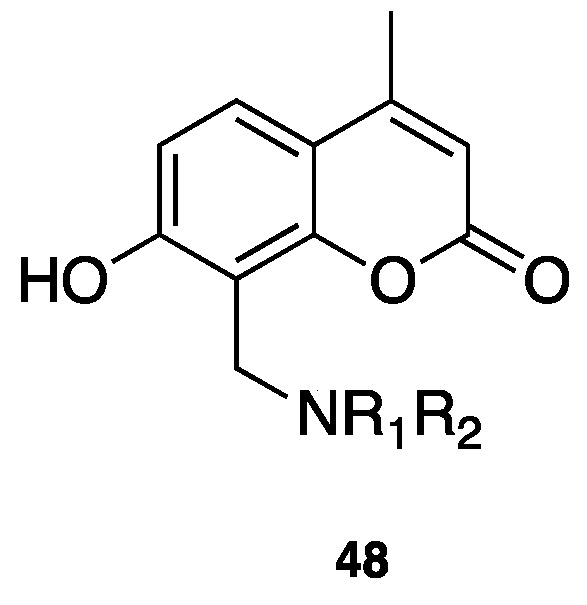
8-Aminomethylcoumarins as multitarget-directed ligands.

More recently, the design of a pool of 7-hydroxy-4,8-disubstituted coumarins by a target-based computational study, including flexible docking on TcAChE and BACE1 models and MD simulations of the interaction with β-amyloid [146], afforded molecules with balanced multiple activities on these targets. Starting from previous results on 8-(aminomethyl)-7-hydroxycoumarins, acting as BACE1 inhibitors, the introduction of bulkier amine substituents (**48**, Figure 32) warranted a fair eeAChE inhibitory activity, with good selectivity over esBChE. Inhibition of self-aggregation of Aβ40 was similar to that of reference compound anthraquinone, while propidium displacement assay suggested a good ability to interfere in AChE-induced amyloid aggregation by binding to the PAS.

**Figure 33:**
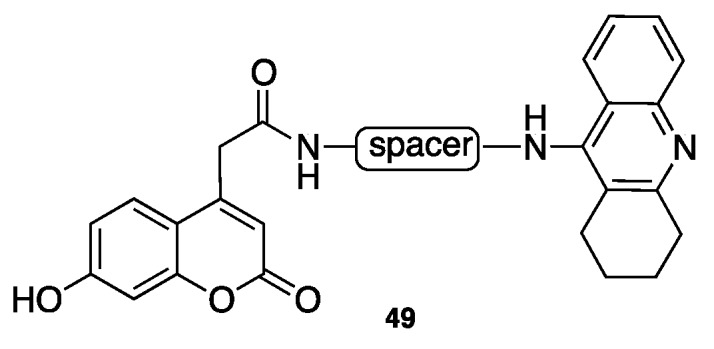
7-Hydroxycoumarin–tacrine conjugates as multitarget-directed ligands.

Targeting reactive oxygen species and metal dyshomeostasis has been pursued by preparing coumarin-based derivatives [147]. An interesting study, still based on conjugates of 7-hydroxycoumarin linked in position 4 to tacrine (**49**, Figure 33), described radical scavenging and Cu(II) chelating properties, besides the desired ChE and β-amyloid aggregation inhibition, leading to the multifaceted biological activity of such class of compounds [148].

**Figure 34:**
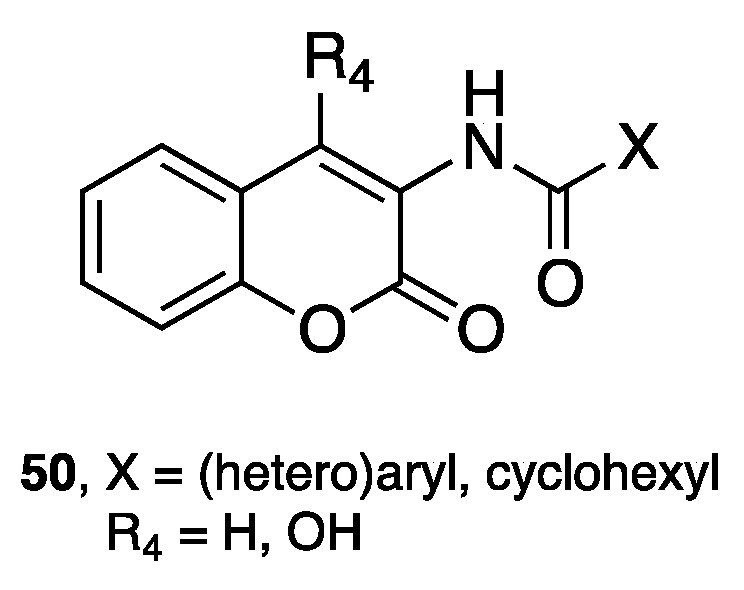
3-Carboxamidocoumarins as multitarget-directed ligands.

Matos and coll. described a series of (hetero)arylamides of 3-aminocoumarin **50** (Figure 34), i.e., a scaffold well known for its MAO inhibitory properties, where the introduction of an antioxidant moiety conferred neuroprotection against oxidative stress [149]. Unfortunately, when the antioxidant moiety (i.e., hydroxyl in position 4) was introduced, a drop of hMAO B affinity followed, while neuroprotection from oxidative insult was unchanged.

### 3.5. Coumarins Acting on Other Targets

The antiproliferative activity of coumarin derivatives is well documented. It was in fact reported that coumarins could act as kinase inhibitors [150], sulfatase inhibitors [151,152,153], selective estrogen receptor modulators [154,155,156], particularly downregulators [157,158,159], 17βHSD3 inhibitors [160] cellular cycle blockers [161,162,163,164,165], and aromatase inhibitors [166,167,168,169,170,171,172,173,174,175]. The latter will be examined in more details herein.

**Figure 35:**
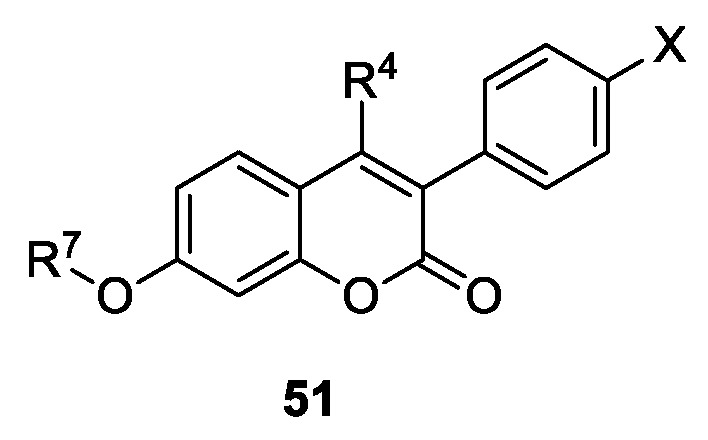
Chen’s coumarin derivatives as aromatase (AR) inhibitors.

Aromatase (AR, CYP19) is a CYP450 enzyme, and its inhibitors examestane, anastrozole, and letrozole are used in the treatment of ER^+^ (estrogen dependent) breast cancer [176]. It was observed that some suitably functionalized coumarins constitute good substrates/inhibitors for some P450 metabolic enzymes [166]. Chen et al. in 2004 prepared some coumarin derivatives that resulted in very potent AR inhibitors [167]. Their coumarin ring presented three points of molecular diversification in position 3, 4, and 7 (**51**, Figure 35), and the best results were obtained when chlorophenyl, benzyl, and methoxy groups were introduced in position 3, 4, and 7 respectively (IC_50_ = 80 nM). Computer modeling studies demonstrated that Chen’s coumarins align very well with the androstenedione, and this could explain their good AR inhibition.

**Figure 36:**
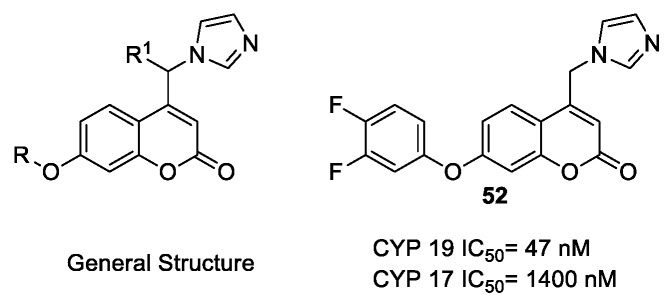
4-Methylimidazolyl coumarins with good inhibitory activity towards AR.

Almost at the same time, our group projected the design and synthesis of new coumarin-based AR inhibitors with good pharmacological, biological, and toxicological profiles. Our first results have been reported in 2004 [168], followed by the publication of a three-dimensional homology model of the human AR enzyme [169] at wwPDB [170], a valuable tool for structure-based design. Our study aimed at the synthesis of a series of 4 (and/or 3)-imidazolylmethyl-7-substituted coumarins as potent and selective AR inhibitors. Many derivatives showed an AR inhibitory potency in the nanomolar range, and high selectivity over 17-*R*-hydroxylase/C17-20 lyase (CYP17). The most potent AR inhibitor was the 7-(3,4-difluorophenoxy)-4-imidazolylmethyl coumarin **52** (Figure 36) with an IC_50_ = 47 nM, a value very close to that of fadrozole (IC_50_ = 52 nM) [171].

Docking studies on CYP19 permitted identification of the most important binding interactions, and the allowed and disallowed regions for appropriate structural modifications of the coumarin core. The coumarin ring establishes one hydrogen bond interaction between the lactone carbonyl and the hydroxyl of Ser478, while the imidazole *N3* binds the iron ion. In addition, good allocation of the phenoxy and benzyloxy groups in a hydrophobic accessory binding site was evidenced. This three-sites interaction model is satisfied when the coumarin ring brings, at position 4, an imidazolylmethyl group, and a phenoxy substituent at position 7.

Compounds from the two studied series (7-benzyloxy and 7-aryloxy derivatives) were selected and tested towards CYP11B1 (steroid 11β-hydroxylase, drug target for Cushing’s syndrome, or metabolic disease) [172] and CYP11B2 (aldosterone synthase, drug target for hyperaldosteronism and congestive heart). The lead of the benzyloxy series (R = Bn, R^1^ = H; Figure 36), was a potent inhibitor of both CYP19 and CYP11B1 (IC_50_ = 0.150 and 0.072 µM, respectively), but showed a lower inhibition for CYP11B2 and for CYP17 (IC_50_ = 0.289 µM and 3% at 2.5 µM, respectively). Among some 7-benzyloxy-substituted congeners, tested against the four different CYPs, the 3′-trifluoromethoxybenzyloxy derivative was a very potent and selective CYP11B1 inhibitor, with an IC_50_ value of 5 nM and 40-, >500- and 30-fold selectivity towards CYP19, CYP17 and CYP11B2, respectively.

**Figure 37:**
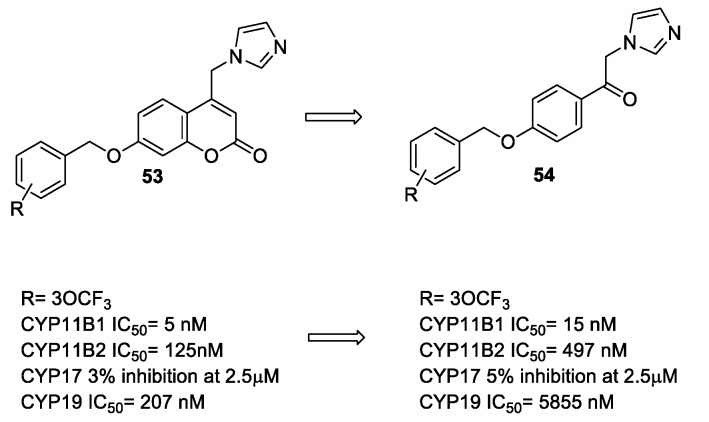
Open analogues of 4-imidazolylmethyl coumarin as selective inhibitors of CYP11B1.

Open analogues **54** of the 7-benzyloxy substituted coumarins **53** (Figure 37) were also prepared. Compound **54**, 2-(1*H*-imidazol-1-yl)-1-(4-{[3(trifluoromethoxy)benzyl]oxy}phenyl) ethanone, was the most potent inhibitor of the series, with a high inhibitory activity towards CYP11B1 (IC_50_ = 15 nM), and an increased selectivity over CYP11B2 (SI = 33) and CYP19 (SI = 390) with respect to the coumarin analogue **53** (R = OCF_3_) [171].

**Figure 38:**
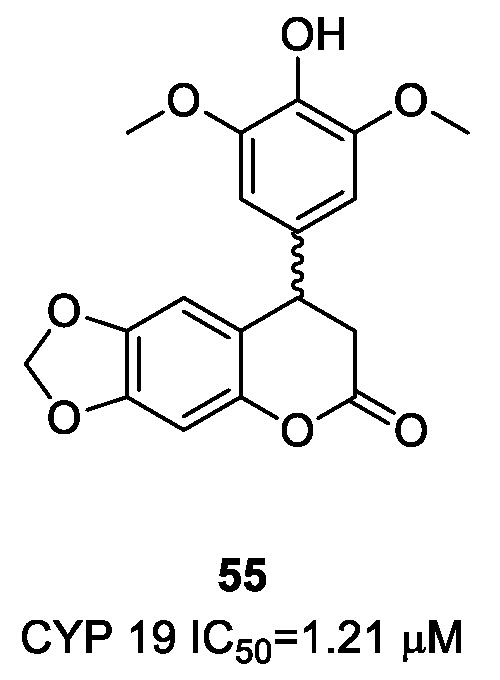
Luqman’s neoflavonoid AR inhibitors.

In 2012, Luqman et al. performed a target-based study on some 4-phenyl substituted 3,4 dihydrocoumarins (neoflavonoids; Figure 38). Monomethoxy, 3,4,5-trimethoxy, and 4-OH substituents on the 4-phenyl ring were favorable for AR inhibition. The most active compound **55** reported in the figure, showed an IC_50_ in the low micromolar range [174].

**Figure 39:**
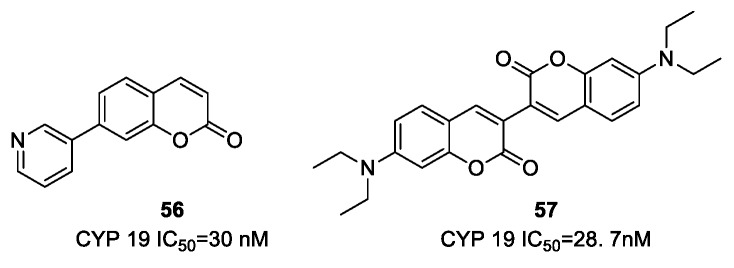
Yamaguchi’s coumarins with AR inhibitory activity.

Very recently, Yamaguchi et al. examined the AR inhibitory effects of 7-diethylaminocoumarin derivatives bearing a substituent at position 3, and additional coumarin derivatives with an amino substituent at position 7 (Figure 39). They found that 7-(pyridin-3-yl)coumarin **56** (IC_50_ = 30.3 nM) and 7,7′-diethylamino-3,3′-biscoumarin **57** (IC_50_ = 28.7 nM) were the most potent AR inhibitors [175].

## 4. Conclusions

Natural and synthetic coumarins have drawn much attention due to their numerous biochemical and pharmacological activities. Industrial interest on coumarins led to the development of cosmetics, agrochemicals, polymeric [2], and optoelectronic materials [3]. Indeed, the largely explored photochemical properties of coumarins have also led to the preparation of fluorescent chemical and biochemical probes, including fluorescent target peptides, proteins, and nucleic acids.

In the present review, we have reported selected studies of a large series of coumarins as versatile, potent, and selective inhibitors of enzymes, and as multipotent ligands addressing NDs. The review has been developed along a research line in the field conceived by our research group.

The effects of substituent type and substitution pattern on the potency and selectivity of the examined coumarins have been discussed, and the proposed SARs analyzed with the purpose to identify the main molecular/structural determinants responsible for the observed activities at the addressed targets. This information may be useful for the design of new coumarins endowed with improved and more selective pharmacological activity. Of course, the chance of success of this design can be highly increased with docking and MD simulations on the selected targets, provided that their three-dimensional structures have been determined by X-ray crystallography or NMR.

As discussed in Section 1.4, the peculiar structure of the coumarin ring determines an easy, and often non-selective, binding to different targets, with which coumarins may establish strong and reversible, polar and apolar interactions, or even irreversible/covalent interactions, as seen for the inhibition of serine proteases illustrated in Figure 6. As a result, the mechanism of action/binding of coumarins to many targets would still deserve further studies, even because multiple mechanisms of action may be operative. This difficult and challenging scenario may be further complicated by the diverse metabolic transformations that coumarins might undergo in vivo, as discussed in Section 1.2. Actually, pharmacokinetic studies in humans indicated an extensive first-pass metabolism of coumarins after oral administration. Therefore, in the design of novel coumarin derivatives addressing known or new targets, particular attention must be devoted to their likely metabolic stability and transformations. The latter can be also profitably used to design molecules that can be activated in vivo through well-known metabolic pathways of coumarin [35,36], and of its substituents [112].

One further issue to take into account is related to the safety of coumarins. Despite their wide diffusion in nature and their significant presence in daily food, concerns have been raised on their true safety. Indeed, contrasting data are coming from literature, and toxic effects have not been studied so profoundly as they would deserve. One reason for the lack of toxicological data is that in vitro and even animal models are inadequate to reproduce the diverse biotransformations of coumarins in humans. However, very luckily, mounting evidence coherently suggests that toxic effects are strongly dose-dependent, and dangerous results only occur at very high doses of coumarins, i.e., difficult to reach in a normal diet. Of course these considerations are applicable to natural coumarins, and cannot be extended to synthetic coumarins, whose toxicity will depend on substituents and substitution partners, and therefore, would deserve dedicated investigations.

In conclusion, despite the new significant findings acquired in the last few decades, the design of potent and selective coumarins, endowed with favorable ADMET properties, especially a higher metabolic stability and lower toxicity, still remain a challenging goal for medicinal chemists, and so coumarins will likely remain on stage for some time, even if no new coumarins have been advanced to the clinical trial since several years.
